# First-Principles Study of Nitrogen Adsorption and Dissociation on PuH_2_ (111) Surface

**DOI:** 10.3390/molecules25081891

**Published:** 2020-04-19

**Authors:** Changshui Wang, Kai Zhang, Peng Song, Xiaofei Hu, Jinglin Mu, Zhichao Miao, Jin Zhou, Hui He

**Affiliations:** 1 Department of Radiochemistry, China Institute of Atomic Energy, Beijing 102413, China; water6377@163.com (C.W.); 15810116091@163.com (K.Z.); songp401@163.com (P.S.); huxiaofei31@163.com (X.H.); 2 School of Chemistry and Chemical Engineering, Shandong University of Technology, Zibo 255000, China; watfros@sdut.edu.cn (J.M.); miaozhichao@sdut.edu.cn (Z.M.)

**Keywords:** density functional theory, molecular and dissociative adsorptions, plutonium mononitride, reaction barrier, PuH_2_ (111) surface, nitrogenation

## Abstract

Plutonium mononitride is one of the main fuels for Generation IV reactors and can be prepared from nitrogenation of plutonium hydride. We investigated the adsorption and dissociation of nitrogen on PuH_2_ (111) surface to elaborate the initial stage of nitrogenation. The adsorption energies varied greatly with respect to the adsorption sites and orientations of the adsorbed molecule. The nitrogen exhibited preferential adsorption above the ccp site, where the molecular nitrogen was nearly parallel to the PuH_2_ surface and pointed to the nearest Pu atom. The orbital hybridization and the electrostatic attraction between the Pu and N weakened the N-N bond in the adsorbed molecule. The mechanism of the dissociation process was investigated within transition state theory, and the analysis of the activation barrier indicated that dissociation of nitrogen is not the rate-determining step of nitrogenation. These findings can contribute to a better understanding of the nuclear fuel cycle.

## 1. Introduction

With the increasing social concern about energy consumption and environmental pollution, nuclear energy has attracted renewed attention in recent years. Therefore, it is crucial to understand the chemical and physical properties of both civilian and weapons materials at various stages of the nuclear fuel cycle. Plutonium is one of the main materials used in nuclear applications, and considerable experimental and theoretical efforts have been devoted to studying the structures and properties of elementary plutonium as well as its compounds [1,2,3,4,5]. For the safety of long-term storage of plutonium, a series of reactions may be involved, such as Pu+x2O2→PuOx, Pu+x2H2→PuHx, Pu+xH2O→PuOx+xH2,  PuHx+12N2→PuN+x2H2 [6,7,8,9,10].

Calculations using density functional theory (DFT) [11,12] have provided a significant opportunity to advance the understanding of Pu-related material structures and reaction mechanisms [3,13,14,15,16]. Huda and Ray presented a useful study of atomic hydrogen adsorption onto δ-Pu (100) and (111) surfaces, and found the preferential adsorption [17]. Their influential investigations of molecular hydrogen interaction with δ-Pu are of great significance, as they compared the dissociation barriers with different surfaces and found the most favorable dissociation channel [18,19]. Goldman’s innovative calculation provided valuable insight into the H_2_ dissociation pathways with an energy map, and also indicated that the dissociation reaction is highly active even at ambient conditions [20,21]. In a thorough study of H_2_ interaction with PuO_2_, Yu and Meng compared various adsorption sites to evaluate H_2_ dissociation and atomic H diffusion energy barriers. Their results indicated that dissociation of H_2_ molecule on PuO_2_ (1 1 0) surface is kinetically favored, while hydrogen permeation through PuO_2_ surface is inhibited [22,23,24]. Sun carried out ab initio molecular calculations and revealed that hydrogen molecules can penetrate into the Pu_2_O_3_ surface [25]. Much of the literature has paid particular attention to the crystal structures and electronic properties of plutonium compounds, and has shown good agreement with experimental results [26,27,28,29,30,31].

Plutonium mononitride (PuN) can be prepared via the nitrogenation of plutonium hydride powder with nitrogen, and is considered fuel for Generation IV reactors, due to the key advantages of high melting point, superior fuel density, and thermal conductivity [32,33,34]. Unlike δ-Pu, PuO_2+x_, and PuH_x_, very little effort has been devoted to studying the physicochemical properties of PuN. In particular, the nitrogenation mechanism of plutonium hydride with theoretical calculations has not been reported. To this end, we performed DFT calculations to investigate the nitrogen adsorption and dissociation on a plutonium dihydride (PuH_2_) surface, i.e., the initial stage of formation of plutonium nitride. Our work aimed to find the most favorable adsorption site and to provide detailed predictions for the nitrogen dissociation channels. These findings should make an important contribution to future experiments and calculations related to the reactions of plutonium-based materials.

## 2. Theoretical Methods and Models

All DFT calculations reported here were carried out with the Cambridge Serial Total Energy Package (CASTEP) [35], implemented in Materials Studio 2019, using Perdew–Burke–Ernzerhof generalized gradient approximation [36], OTFG norm-conserving pseudopotentials [37], and a plane wave basis set [38] with a cut-off energy of 800 eV. The reference atomic valence configurations used were H (1s^1^), N (2s^2^2p^3^), and Pu (5f^6^6s^2^6p^6^7s^2^). Periodic boundary conditions were applied, and the Monkhorst–Pack k-points for the Brillouin zone [39] were tested to ensure the validity of the results. The geometries of the system were relaxed until the residual forces were less than 0.04 eV/Å and the total energy was less than 1.0 × 10^−5^ eV on each atom. Spin–orbit coupling was neglected, as in the recent work of Tegner [40] and Kaltsoyannis [41], because spin−orbit coupling has a small effect on the surface stability [42] and reaction barriers [21] of plutonium-based materials.

The face-centered cubic (fcc, fluorite type) structure of bulk PuH_2_ was first optimized without spin polarization and the calculated lattice parameter *a* was 5.19 Å, which was smaller than the experimental value in a number of respects. Spin-polarized DFT calculations with the antiferromagnetic order [31] predicted better agreement with the lattice parameter of 5.33 Å. To accurately account for the effective correlation between f-orbitals, DFT + *U* calculations were carried out (on-site Hubbard correction *U*, 2.0 eV) and the lattice parameter *a* was 5.40 Å, which was in good agreement with the previous experimental value, 5.395 Å [43]. The Hubbard *U* parameter value was consistent with Ao and Gao’s work, in which this value was tuned to generate a lattice parameter fully accordant with experimental observations [44]. Although the widely-accepted Hubbard correction for describing Pu is 4.0 eV [28,45,46], this value slightly overestimates the lattice parameter of PuH_2_ [21]. Additionally, Goldman has reported much smaller values and demonstrated the *U* values have little effect on the dissociation chemistry [21]. These reports indicate that there was no need to evaluate the choice of *U* values, and the effects of Hubbard correction were not discussed in the present study.

**Figure 1:**
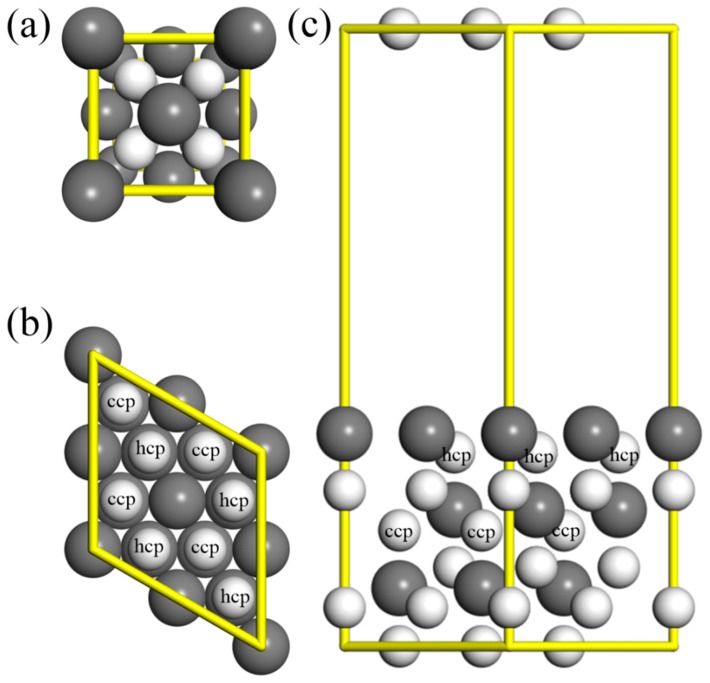
Calculated structures of PuH_2_ (**a**) unit cell, and (**b**) top and (**c**) side views of the PuH_2_ slab. The gray and white spheres represent Pu and H atoms, respectively. The H atoms labeled as hcp/ccp are above the middle/ bottom layer Pu atoms.

After optimization of the bulk structures, a PuH_2_ (111) slab is created with three layers of fcc PuH_2_ and each layer contained four PuH_2_ structural motifs, as shown in Figure 1. The choice of three layers was quite satisfactory considering that the adsorbed molecule should not interact with atoms beyond the first three layers and the surface energies of δ-plutonium converge within the first three layers [5,19,47]. The vacuum was set to 16 Å in the <001> direction and plutonium atoms were exposed to the surface. Calculations with hydrogen atoms exposed on the surface were not taken into account, because these exposed hydrogen atoms were located above the Pu layer and exhibited a high degree of surface mobility without forming the hydride product [17,21]. The plutonium and hydrogen atoms of the bottom layer were frozen in the positions of their crystal lattice while other atoms were allowed to relax in the optimization of the slab. Following this, the adsorption and dissociation of nitrogen were investigated.

To further verify the reliability and effectiveness of our calculation, the adsorption of hydrogen atom on the δ-Pu (111) surface was investigated with the same parameters. The adsorption energies of the ccp and hcp site were −2.57 and −2.61 eV, which were consistent with the previous results, −2.487 and −2.509 eV [21].

## 3. Results and Discussion

We first discuss the adsorption of molecular nitrogen onto PuH_2_ surfaces, then focus on the situation of atomic nitrogen, and finally provide the most favorable process for nitrogen dissociation.

### 3.1. Molecular Adsorption

**Figure 2:**
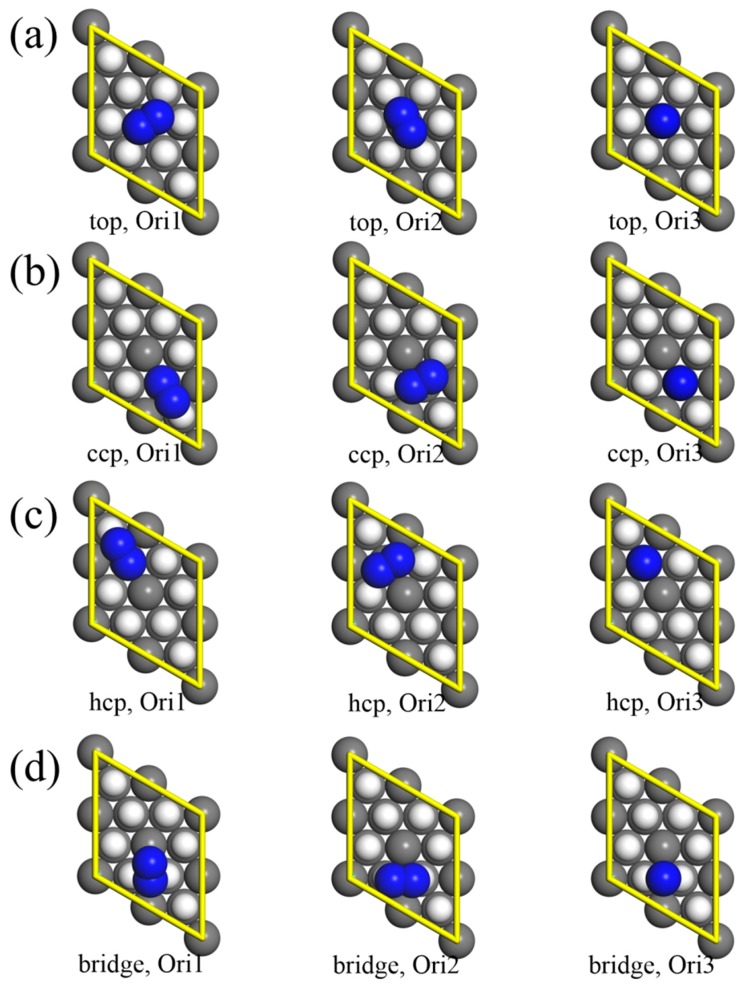
Molecular nitrogen adsorption onto PuH_2_ (111) surfaces with different sites and orientations: (**a**) top, (**b**) ccp, (**c**) hcp, and (**d**) bridge site. The gray, blue, and white spheres represent Pu, N, and H atoms, respectively.

The adsorption of molecular nitrogen onto PuH_2_ surfaces was investigated, and the optimized geometries with different adsorption sites and orientations are shown in Figure 2. The four different adsorption sites were (i) directly above a surface plutonium atom (top site); (ii) on the middle of two nearest-neighbor surface plutonium atoms (bridge site); (iii) at the hollow site on top of a Pu atom on the second layer (hcp site); and (iv) at the hollow site on top of a Pu atom on the third layer (ccp site). For each of these sites, we considered three different orientations of N_2_ molecule for adsorption: (i) parallel to the surface and pointing to the nearest Pu atom (Ori1); (ii) parallel to the surface and vertical to Ori1 (Ori2); and (iii) vertical to the surface (Ori3). These adsorption sites and orientations of N_2_ molecule were the same as the calculations with δ-plutonium (111) surface, and the plutonium and hydrogen atoms were frozen in their optimized slab positions [21,47,48]. Following the geometry optimizations with spin polarization, the adsorption energies were then calculated from the following equation.

(1) $$E_{ads}=E\left({\mathrm{PuH}}_{2}-\mathrm{slab}+N_{2}\right)-E\left(N_{2}\right)-E\left({\mathrm{PuH}}_{2}-\mathrm{slab}\right)$$

where $E\left({\mathrm{PuH}}_{2}-\mathrm{slab}+N_{2}\right)$, $E\left(N_{2}\right)$, and $E\left({\mathrm{PuH}}_{2}-\mathrm{slab}\right)$ are the total energies of the PuH_2_ slab with adsorbed N_2_, N_2_ molecule in gas phase, and the PuH_2_ slab, respectively. The probability of adsorption can thus be suggested by the magnitude of the absolute value of the adsorption energy. The adsorption energies and the optimized adsorption parameters for different adsorption sites and orientations are provided in Table 1. The Brillouin zones of the slabs were sampled by 2 × 2 × 1 k-point meshes, and increasing the k-point meshes to 3 × 3 × 1 changed the total energy and adsorption energy by only 0.001 eV/atom and 0.01 eV. The distances r_N_ shown in the table are the N-N distances in the adsorbed N_2_ molecule, while the distances r_d_ are the vertical distance from the PuH_2_ surfaces to the closest N atoms.

There was no obvious change in the adsorption of the top site compared with the predesigned structures. The molecular nitrogen exhibited less favorable adsorption at this site than the other three sites. The adsorption parameters of the two parallel orientations were almost identical. The adsorption energy of Ori2 orientation was only 0.01 eV lower than that of Ori1 orientation, while the distance r_d_ was 0.02 Å closer. The bond lengths r_N_ were both slightly stretched from the theoretically optimized results 1.10 Å. In the case of vertical orientation, the N_2_ molecule showed shorter vertical distance to the PuH_2_ surface, and adsorption energy also increased accordingly. Similar results have been reported in previous studies of oxygen adsorption on Pu (111) surface, and the reasonable explanation for this is that the adsorbate interacts with only one plutonium atom [47].

Significant differences were found in the adsorption of ccp site. The Ori1 orientation revealed the lowest adsorption energy, and was the most stable configuration. In this orientation, N_2_ was very close to the PuH_2_ surface, and the bond length was strongly stretched. Additionally, the orientation of the nitrogen molecule was also tilted. One nitrogen atom moved towards the surface, and almost completely occupied the ccp site. The other nitrogen atom was slightly further from the surface, and the nitrogen molecule deviated from the original adsorption position. Finally, the angle of the nitrogen molecule to the surface was about 19°. It was inferred from these results that the ccp site had a low adsorption energy with the nitrogen atom. The Ori2 orientation also exhibited strong chemisorption, with the distance r_d_ of 1.48 Å and bond length r_N_ of 1.25 Å. Unlike Ori1 orientation, Ori2 remained almost parallel to the surface. There were similarities between the ccp site and top site expressed by adsorption energies of vertical orientation, but the former provided longer N-N bond lengths. This adsorption energy seemed inconsistent with the above result that nitrogen atoms were strongly adsorbed at ccp site. However, the nitrogen atom further from the surface was less affected, and revealed the opposite attraction to closer nitrogen atom, resulting in weak adsorption. According to these data, it could conceivably be hypothesized that no vertical adsorption would be strong chemisorption.

The adsorption parameters and the configurations of molecular nitrogen in the hcp site with different orientations presented similarly to those of the ccp site. The Ori1 orientation revealed the lowest adsorption energy and the shortest vertical distance, while the Ori3 orientation offered the opposite adsorption capacity and bond length. The results reported in Table 1 also suggest that the adsorption capacity of hcp site was between that of the ccp site and the top site.

For case of the bridge site, Ori2 was the preferred orientation. Although the center of the molecular nitrogen was placed at the bridge site, there were noticeable variations in the chemical environments of the two nitrogen atoms. One nitrogen atom was close to the ccp site, while the other was near the hcp site. Drastic changes took place in the adsorption position and orientation of the nitrogen molecule due to the difference of adsorption capacity between the two sites, resulting in the adsorption of the ccp site with Ori1 orientation. Thus, the X and Y directions of the nitrogen molecule with Ori2 orientation were fixed, and the adsorption parameters are shown in Table 1. The Ori1 orientation was also no longer parallel to the surface, and the two nitrogen atoms coordinated with different Pu atoms. The adsorption energy was −0.65 eV, and N-N bond was extended to 1.17 Å.

To further evaluate the dispersion corrections, we performed calculations using the DFT-D3 method [49], based on the optimized configurations. The adsorption energies contributed by the inclusion of the corrections are denoted as E_D3_, as shown in Table 1. The adsorption energies decreased by 0.18–0.21 eV due to the D3 dispersion corrections, and the order of adsorption energies remained unchanged, suggesting little dependence between the adsorption sites and orientations. These results were similar to Kaltsoyannis’s work, in which the adsorption of water onto PuO_2_ was investigated [50].

In all these cases, the adsorption was molecular in nature, and there were similarities between the attitudes expressed by the adsorption configurations in this study and those described by Ray in the investigation of H_2_ interaction with the Pu [19]. The adsorption energies varied greatly with respect to the various adsorption sites and the orientations of nitrogen molecules, and suggest a moderate adsorption capacity compared with the adsorption of H_2_ and O_2_ onto Pu surface [19,47]. We also observed that for almost all the cases, the adsorption energy and the bond length were shown to vary in response to each other, while noticeable discrepancy was found in the vertical distance r_d_. This inconsistency may be explained by the chemical environments of the different nitrogen atoms. In general, the adsorption of nitrogen onto the PuH_2_ surface was exothermic, and the most stable geometry was at the ccp site, where the molecular nitrogen was nearly parallel to the surface and pointed to the nearest Pu atom. The suboptimal adsorption site was hcp with a similar orientation.

**Figure 3:**
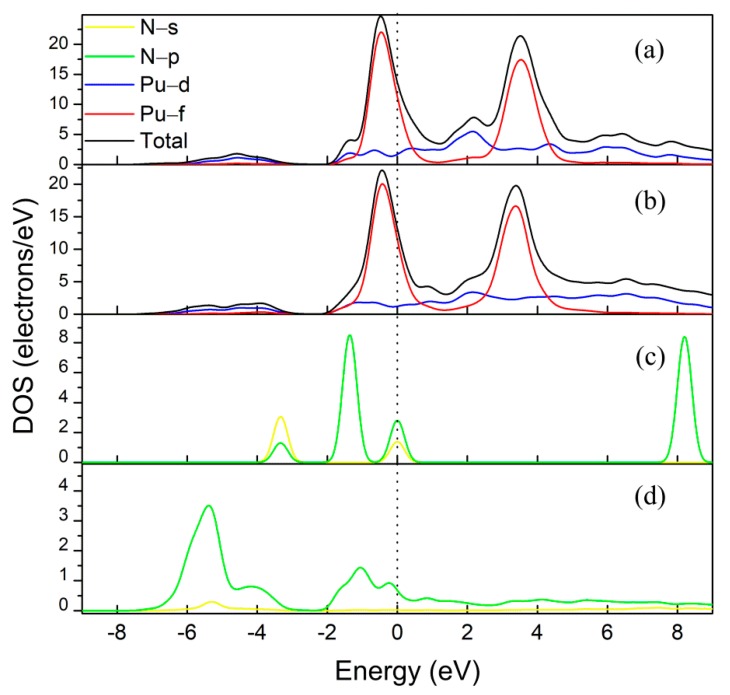
Partial density of states for (**a**) clean PuH_2_ (111) surface; (**b**) PuH_2_ (111) surface with adsorbed N_2_; (**c**) gaseous N_2_, nitrogen; and (**d**) adsorbed N_2_. The smearing width was set to 0.2 eV and the Fermi levels were set at 0.

Figure 3 plots the partial density of states (PDOS) of the PuH_2_ (111) surfaces with and without nitrogen adsorption. For the sake of brevity, only the most favorable nitrogen-adsorbed case (ccp Ori1) is shown. The smearing width was set to 0.2 eV and the Fermi levels were set at zero. It was clear that the main peak of the PuH_2_ (111) surface at the Fermi level was dominated by the Pu−5f orbitals, while the main peak of N_2_ at the Fermi level was a combination of N-2s and N-2p. Variations appeared in the Pu-6d orbitals near the Fermi level upon nitrogen adsorption, and the Pu-5f orbitals remained nearly undisturbed. The PDOS of N_2_ revealed that the valence states of N (2s and 2p orbitals) shifted to the lower levels. Moreover, the peak above the Fermi level disappeared. These results suggest that hybridizations took place between the Pu-6d and N-2p orbitals, and that the antibonding orbitals in N_2_ accepted electron density transferred from Pu-6d orbitals. Similar results were also described by Hirshfeld [51] charge analysis, which provided evidence that N_2_ molecules gain electrons form PuH_2_ slab, revealing that the ionic aspect of Pu-N bonding plays a significant role in the adsorption product.

### 3.2. Dissociative Adsorption

**Figure 4:**
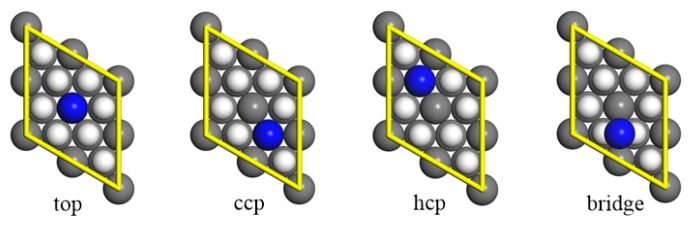
Atomic nitrogen adsorption onto PuH_2_ (111) surfaces with different sites: top, ccp, hcp, and bridge site. The gray, blue, and white spheres represent Pu, N, and H atoms, respectively.

To further illustrate the adsorption capacity and predict the dissociation process of the nitrogen molecule on the PuH_2_ (111) surface, the adsorption of atomic nitrogen was investigated. The four specific adsorption adsorption sites were top, bridge, ccp, and ccp site, as presented above. After the geometry optimizations, the adsorption energies were then evaluated from

(2) $$E_{ads}=E\left({\mathrm{PuH}}_{2}-\mathrm{slab}+\mathrm{nN}\right)-E\left({\mathrm{PuH}}_{2}-\mathrm{slab}\right)-n\times E\left(N\right)$$

where $E\left({\mathrm{PuH}}_{2}-\mathrm{slab}+\mathrm{nN}\right)$, $E\left({\mathrm{PuH}}_{2}-\mathrm{slab}\right)$, and E(N) are calculated with spin polarization, and n represents the number of nitrogen atoms. The chemisorption energies and the equilibrium geometries of the N atom to the PuH_2_ surface are given in Table 2, and Figure 4, respectively.

**Figure 5:**
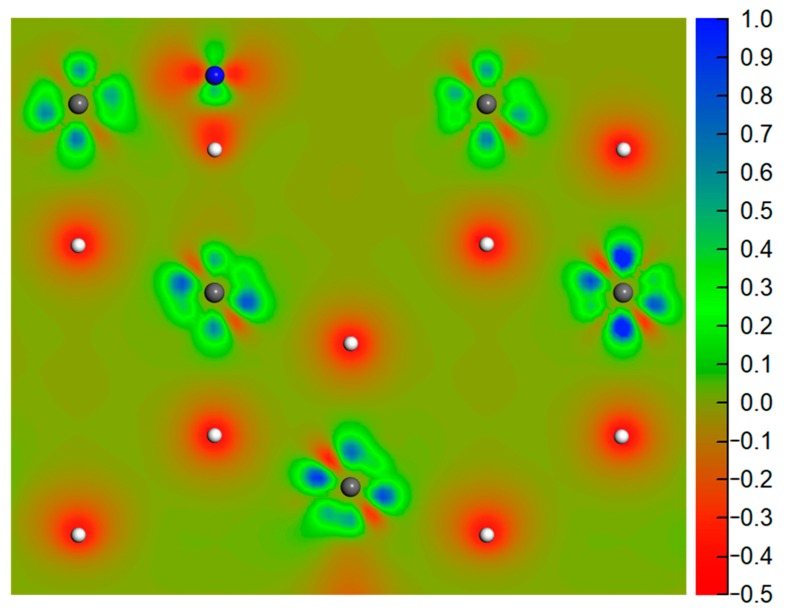
Charge density difference contour map along the (110) plane. The loss of electrons is indicated in blue, while electron enrichment is indicated in red. The gray, blue, and white spheres represent Pu, N, and H atoms, respectively.

The nitrogen exhibited preferential adsorption above the ccp site, closely followed by the bridge and hcp sites. For the ccp site, spin polarized calculation indicated an adsorption energy of −6.85 eV with a vertical distance of 0.45 Å. The nitrogen atom embedded itself in the surrounding plutonium atoms, yielding three Pu-N bonds, and the average bond lengths were about 2.23 Å. In the case of the two-fold coordinated bridge site, adsorption was slightly less favored than at the ccp site. The nitrogen experienced the most unfavorable adsorption at the top site, where the nitrogen atom was bonded to only one Pu atom. These results suggest that the adsorption energies strongly depend on the number of Pu-N bonds. However, this conclusion cannot be applied to the hcp site. Similar Pu-N bonds were found at the ccp and hcp sites, while their adsorption energies differed by more than 1 eV. This rather contradictory result may be interpreted in terms of the electronic structures of PuH_2_. The ionic character of Pu-H bond was investigated by Hirshfeld analysis, revealing that electrons were transferred from Pu to H atoms. When a nitrogen atom with a larger electronegativity was adsorbed directly above the hcp H atom (1.18 Å), it gained −0.43 e from the PuH_2_ slab and formed strong Coulombic repulsion with the hcp H atom, resulting in less favored adsorption than at the ccp site. To further reveal the bonding characteristics in this adsorption site, we plotted the charge density difference contour map along the (110) plane, as shown in Figure 5. The ionic nature of the Pu−H bond was clearly revealed, while abnormal charge density was found in the direction of the axis of the N-H bond, suggesting strong repulsion between N and H atoms.

Following the calculations of the adsorption of the nitrogen molecule and a single nitrogen atom, dissociative adsorption was investigated to explore the nitrogen dissociation process. Since the ccp site had the largest adsorption capacity, one of the dissociated nitrogen atoms was assumed to occupy this position. We then focused on the question of which site was preferred by the other nitrogen atom. Based on the above discussion, the top site was excluded for its instability compared with other adsorption sites, and the final adsorption geometries were determined as follows: (i) both atoms were at ccp sites (ccp-ccp site); (ii) one atom was at a ccp site, and the other was at a bridge site (ccp-bridge site); (iii) one atom was at a ccp site and the other was at the ortho hcp (ccp-hcp1 site); (iv) one atom was at a ccp site and the other was at the para hcp (ccp-hcp2 site).

The adsorption and dissociative adsorption energies with related parameters are shown in Table 3. The dissociative adsorption energies are defined as $E_{dis}=E\left({\mathrm{PuH}}_{2}-\mathrm{slab}+\mathrm{nN}\right)-E\left({\mathrm{PuH}}_{2}-\mathrm{slab}+N_{2}\right)$. The results indicated that the dissociative adsorption of ccp site molecular nitrogen was exothermic, and the ccp-ccp site was the most favorable dissociative adsorption site. In fact, the adsorption energy of the ccp-ccp site was twice that of the ccp site when a single nitrogen atom was adsorbed, indicating little interaction between the two nitrogen atoms. Nearly identical vertical distances and bond lengths of Pu-N also clearly supported and confirmed this conclusion. For the case of ccp-bridge site, the bridge site atom was not stable and fixed, similarly to the case of molecular adsorption at bridge site and Ori2 orientation. Finally, we compared the adsorption energies of the two ccp-hcp sites. The nitrogen atom exhibited slightly less preferred adsorption at the ortho hcp site. This relationship may be interpreted by the repulsion of the two negatively charged nitrogen atoms. Additional evidence arises from the fact that the distance between two nitrogen atoms in ccp-hcp1 was a little larger than that of the exact ccp-hcp. Furthermore, noticeable discrepancies were also found in the adsorption energies between dissociative adsorption and two monoatomic adsorption. All these results suggest that the ccp-ccp site was most favorable dissociative adsorption site, and the ratio of nitrogen atom to plutonium atom was 1:1. This result is consistent with the previous literatures where only one stoichiometric plutonium nitride was formed [32].

**Figure 6:**
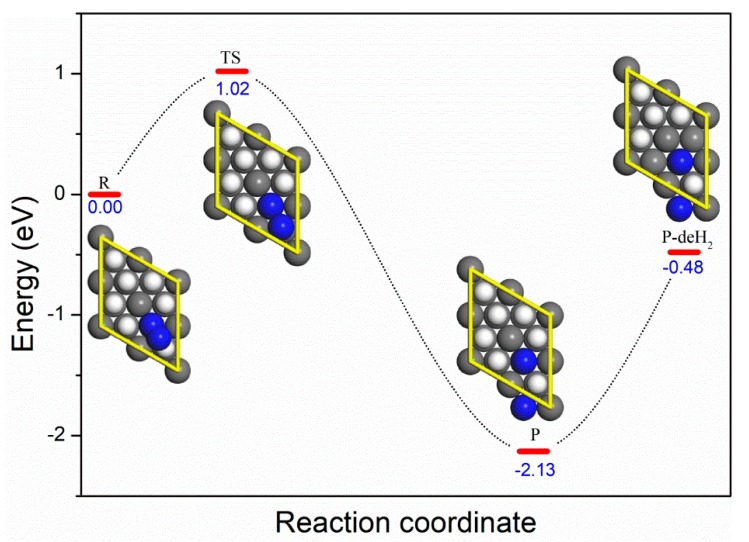
The potential energy profile of the nitrogen dissociation. The preferential adsorption of molecular nitrogen with the ccp site and Ori1 orientation was chosen as the starting point R, and the ccp-ccp was the product P. P-deH_2_ represents the dehydrogenation product.

According to these data, we performed a transition state search with the complete linear synchronous transit/quadratic synchronous transit algorithm [52]. To avoid complicated and worthless calculations, the preferential adsorption of molecular nitrogen with the ccp site and Ori1 orientation was treated as our starting point (R) of the dissociation channel, considering its large adsorption energy and high probability. The potential energy profile is shown in Figure 6. Following the reaction coordinate, the activation barrier for the dissociation process was 1.02 eV, and the dissociated nitrogen atoms were located at the ccp-ccp site, forming the product P. The reaction path was confirmed by nudged elastic band (NEB) method, and similar N_2_ dissociation results have been reported on a body-centred cubic Fe surface with a higher reaction energy barrier [53]. Our results indicate that the adsorption and dissociation of nitrogen onto PuH_2_ (111) surface is energetically favorable, and the strong chemisorption weakens the N-N bond, allowing the dissociation to take place even at room temperature. Muromura and Ouchi suggest that the rate-determining step of nitrogenation of plutonium hydride is the dehydrogenation process rather than the dissociation of nitrogen [8]. To further illustrate this issue, the dehydrogenation products (P-deH_2_) were optimized and the calculations suggested a large energy penalty (1.6 eV) for dehydrogenation. Our theoretical calculations are consistent with the experimental results.

## 4. Conclusions

In the present work, we investigated the adsorption and dissociation of nitrogen onto PuH_2_ (111) surface. Noticeable variations in the adsorption energies were shown in the molecular adsorption, and the most favorable adsorption took place at a ccp site, where the molecular nitrogen was nearly parallel to the PuH_2_ surface and pointed to the nearest Pu atom. Both the orbital hybridization and the ionic electrostatic attraction played significant roles in the adsorption product, and the nitrogen-nitrogen bond was greatly weakened. Atomic nitrogen also exhibited preferential adsorption above the ccp site and the dissociative adsorption was strongly exothermic. The mechanism of the dissociation and diffusion process of nitrogen was investigated by transition state search with the complete synchronous transit/quadratic synchronous transit algorithm. The relatively low activation barrier indicated that this dissociation can take place even at ambient temperature. These results also suggest that the rate-determining step of nitrogenation is the dehydriding process rather than dissociation of nitrogen, and future experiments and calculations should devote their efforts to the dehydrogenation process. Further attention should also be paid to the acceleration of dehydrogenation, such as via temperature, pressure, and catalyst, to accelerate the nitrogenation of plutonium hydride. Our calculations represent the initial stage of formation of plutonium nitride, and further studies of the nitrogenation of plutonium hydride are currently underway.

## Figures and Tables

**Table 1:** Adsorption parameters of N_2_ adsorption on PuH_2_ (111) surface. The parameter r_d_ is the vertical distance from the surface to the closest N atoms, and r_N_ is the N-N distance in the adsorbed N_2_ molecule. E_D3_ denotes the increase of the adsorption energies with the inclusion of the D3 corrections.

Site.	Orientation	E_ads_ (eV)	E_D3_ (eV)	r_d_ (Å)	r_N_ (Å)
top	Ori1	−0.20	−0.18	2.60	1.14
	Ori2	−0.21	−0.18	2.58	1.15
	Ori3	−0.49	−0.16	2.50	1.13
ccp	Ori1	−1.63	−0.21	0.93	1.34
	Ori2	−0.94	−0.19	1.48	1.25
	Ori3	−0.54	−0.19	1.37	1.19
hcp	Ori1	−0.96	−0.19	1.25	1.28
	Ori2	−0.84	−0.18	1.56	1.24
	Ori3	−0.31	−0.18	1.79	1.15
bridge	Ori1	−0.66	−0.19	1.77	1.17
	Ori2 ^1^	−0.89	−0.19	1.29	1.25
	Ori3	−0.37	−0.19	1.72	1.16

^1^ The X and Y directions of the nitrogen molecule with this orientation are fixed.

**Table 2:** Adsorption energies of atomic nitrogen adsorption onto PuH_2_ (111) surface. Adsorption parameter r_d_ is the vertical distance from the surface and and r_Pu-N_ is the average distance of Pu-N.

Site	E_ads_ (eV)	r_d_ (Å)	r_Pu-N_ (Å)
ccp	−6.85	0.45	2.23
top	−4.26	1.81	1.81
bridge	−5.85	0.96	2.11
hcp	−5.67	0.45	2.22

**Table 3:** Adsorption and dissociative adsorption energies of the possible dissociative adsorptions of nitrogen onto PuH_2_ (111) surface. Adsorption parameter r_d_ is the vertical distances from the surface and r_Pu-N_ is the average distances of Pu-N. The subscript A denotes the ccp site atom, and B denotes the other.

Site	E_ads_ (eV)	E_dis_ (eV)	r_d-A_ (Å)	r_Pu-N-A_ (Å)	r_d-B_ (Å)	r_Pu-N-B_ (Å)
ccp-ccp	−13.60	−2.13	0.45	2.23	0.46	2.23
ccp-bridge ^1^	−12.48	−1.01	0.42	2.22	0.94	2.10
ccp-hcp1	−11.86	−0.39	0.33	2.20	0.90	2.39
ccp-hcp2	−12.17	−0.70	0.51	2.24	0.54	2.24

^1^ The X and Y directions of the bridge nitrogen atom were fixed.
